# Steroid hormone regulation of immunometabolism and inflammation

**DOI:** 10.3389/fimmu.2025.1654034

**Published:** 2025-09-17

**Authors:** Ley Cody Smith, Mohankumar Ramar, Gregory L. Riley, Clinton B. Mathias, Ji-Young Lee

**Affiliations:** ^1^ Department of Pharmaceutical Sciences, University of Connecticut School of Pharmacy, Storrs, CT, United States; ^2^ Department of Nutritional Sciences, University of Connecticut, Storrs, CT, United States

**Keywords:** steroid hormones, immunometabolism, immune, inflammation, glycolysis, oxidative phosphorylation

## Abstract

The metabolism of immune cells adapts to support the energy demands for their activation, differentiation, and effector functions through a process known as metabolic reprogramming. This metabolic plasticity is influenced by both extrinsic and intrinsic factors, including steroid hormones such as glucocorticoids, androgens, progestogens, and estrogens. These critical mediators modulate immune function and inflammatory responses through genomic and non-genomic regulation of intracellular metabolic pathways, including glycolysis, the tricarboxylic acid cycle, and oxidative phosphorylation. Interestingly, these effects appear to be dependent on cell type, hormonal concentration, and microenvironmental context. Herein, we discuss how steroid hormones regulate inflammation and immunometabolism and summarize recent studies highlighting immunometabolic regulation by steroid hormones as the key driver of their immunomodulatory effects. We also address potential mechanisms contributing to their seemingly dichotomous and context-specific regulation. Understanding the link between steroid hormone signaling, immunometabolism, host defense, chronic inflammation, and immunity will expand our understanding about how biological sex and stress influence the immune system and facilitate more precise therapeutic targeting of immune cell activity to mitigate inflammation- and immune-mediated diseases.

## 1 Introduction

Immune cells modify the metabolic pathways by which they generate energy to appropriately respond to sterile and infectious stimuli (1). This metabolic reprogramming is triggered by the activation of pattern recognition receptors and cytokine receptors that initiate signaling cascades and activate diverse transcriptional regulators that induce or inhibit the expression of substrate uptake transporters, enzymes, and mitochondrial proteins. In turn, this shifts energy production between aerobic glycolysis, the TCA cycle, and oxidative phosphorylation (2). Changes in metabolism can also modulate immune cell activity, as highlighted by numerous studies reporting that modifying substrate availability or utilization can influence immune cell proliferation, differentiation, activation, and effector functions (3). Thus, understanding the intricate and bidirectional relationship between intracellular metabolism and immune cell activation and function is essential to develop targeted therapeutic strategies to modulate immune and inflammatory responses and mitigate disease.

Immune cell activation and function are regulated by sex- and environmental-based factors. For example, males and females respond to foreign and self-antigens differently, and these responses may change over the course of the lifespan, highlighting gonadal sex hormones as major immunoregulatory agents. In this regard, females exhibit increased susceptibility to autoimmune disease development whereas males show increased susceptibility to non-reproductive malignant cancers (4). Environmental factors such as stress are also known to affect susceptibility to illness by modulating the immune system, sometimes in a sexually dimorphic manner (reviewed in 5 and 6). Underpinning these sex- and environmental-based differences are the actions of steroid hormones, including estrogens, progestogens, androgens, and glucocorticoids. The steroid hormones are derived from cholesterol and regulate reproduction, stress responses, metabolism, and immunity (7). Their levels fluctuate following circadian and ultradian rhythms throughout the estrus (in rodents) and menstrual (in humans) cycle, as well as throughout the lifespan (8–12).

Steroid hormones signal through their cognate steroid hormone receptors, NR3 class nuclear receptors that share similar structures, including an N-terminal transactivation domain, a highly conserved central DNA binding domain, and a C-terminal ligand-binding domain (13). Upon ligand binding, the receptors undergo conformational changes that facilitate the recruitment of co-regulatory proteins and their translocation to the nucleus, where they interact with hormone response elements (HREs) to regulate gene transcription (13). In addition to these direct genomic mechanisms, some steroid hormone receptors (e.g., estrogen receptors and the glucocorticoid receptor) can indirectly regulate gene expression at distinct promoters by tethering to other transcription factors (e.g., AP-1 and NF-κB) (14, 15). The steroid hormones also signal through non-genomic mechanisms initiated at the cell membrane by palmitoylated steroid hormone receptor variants or GPCRs (e.g., G-protein coupled estrogen receptor 1, GPER1); these receptors activate kinase pathways (e.g., MAPK/ERK and phosphoinositide 3-kinase [PI3K]/protein kinase B [Akt]) and modulate gene expression by regulating the activity of the cytoplasmic nuclear receptors and growth factor receptors (16).

Several reviews have thoroughly described the roles of steroid hormones in regulating intracellular metabolic pathways and immune cell development, activation, and function separately, but they rarely connect the two (17–21). Interestingly, these effects appear to vary depending on the cellular microenvironment, cell type, disease or life stage, and concentration of ligand. Parallel to these discordant responses on immune cells are corresponding changes in intracellular metabolism. Thus, the purpose of this review is to explore the interaction between metabolism and immune regulation with a focus on steroid hormone modulation as a key mediator of these seemingly dichotomous effects. This review is focused on immune cells which have been observed to exhibit metabolic reprogramming and be regulated by steroid hormones; these include T cells, macrophages, myeloid-derived suppressor cells (MDSCs), and dendritic cells.

## 2 Metabolic reprogramming in immune cells

Immune cells play a pivotal role in protecting us from disease and in maintaining tissue integrity during conditions of homeostasis. The rapid activation and resolution of immune and inflammatory responses, accompanied by the secretion of numerous proteins, lipids, and other mediators, incur a significant bioenergetic cost requiring the increased uptake of glucose, fatty acids, and amino acids. This often requires a shift in the metabolism of immune cells corresponding to the state they are in, i.e., quiescent, effector, memory, or regulatory. Each of these states can present with a different metabolic phenotype.

### 2.1 T cells

T cells are the major cellular mediators of the adaptive immune system and comprise diverse subpopulations with unique effector functions (22). Both T cell development and function are marked by distinct stages that are associated with changes in morphology and phenotype. Furthermore, the induction of an immune response is accompanied by rapid shifts in T cell status including naïve, activated, effector, and memory phenotypes that come at a significant bioenergetic cost to the cell. Metabolic reprogramming fuels the various stages allowing T cells to maintain optimal metabolic fitness and prime for maximum responsiveness during states of heightened immune activity (23). In T-cell development, elevated expression of hypoxia-inducible factor 1-alpha (HIF-1α) leads to increased induction of glucose transporter type 1 (GLUT1) and enhanced glycolysis and flux through the pentose phosphate pathway to support β-selection (24–26). Then, CD4 or CD8 selection results in a sharp reduction in glucose metabolism and shift to mitochondrial oxidative phosphorylation through down-regulation of HIF-1α (26). This shift is essential for the maturation of memory-phenotype T cells (27).

Naïve or quiescent T cells have minimal metabolic needs and maximize the glycolysis and oxidative phosphorylation pathways by fully oxidizing glucose and generating ATP. The predomination of these pathways provides these cells with the energy required to conduct immune surveillance (1). In contrast, the processes of T cell activation, clonal expansion, differentiation into various effector T cell subsets, and subsequent effector function are characterized by increased energetic demands, resulting in the successive generation and replenishment of biosynthetic precursors that power rapid cell growth and proliferation (23, 28). In CD4+ cells, Th1 and Th17 activation is associated with increased expression of glutamine transporters, which provides more substrates for the TCA cycle as well as enhanced fatty acid biosynthesis (29–31). On the other hand, Th2 and T regulatory cell (Treg) differentiation and activation involve transitions to fatty acid oxidation for energy acquisition (30, 32). In CD8+ T cells, increased expression of GLUT1, lactate dehydrogenase, and hexokinase and a concomitant reduction in carnitine palmitoyltransferase 1A (CPT1a) expression shifts metabolism from fatty acid oxidation to glycolysis, supporting the synthesis of nucleotides and serine for activation (33).

At the end of an immune response, the cells that survive to become memory T cells revert back to lipid oxidation with an increased capacity for efficient energy generation. Because memory T cells do not proliferate or produce cytokines, they have a similar metabolic profile as naïve T cells, wherein fatty acid oxidation and oxidative phosphorylation are sufficient to sustain T cell survival and to meet their energetic requirements. However, unlike naïve T cells, they have increased spare respiratory and glycolytic capacities, with elongated mitochondria and increased expression of electron transport chain proteins. As such, memory T cells exist in a state of improved metabolic fitness where they are ready to engage pathogens upon antigen stimulation (34).

### 2.2 Macrophages

Macrophages are innate immune cells that play important regulatory roles in various tissues and are essential in host defense. They comprise diverse subpopulations characterized by their ontogeny and polarization state (35). The subpopulations are broadly classified into two phenotypes: pro-inflammatory/M1 that promotes Th1-mediated responses against intracellular bacteria with increased phagocytic activity, and an anti-inflammatory/M2 phenotype that promotes type 2 inflammation (36). Macrophages undergo metabolic reprogramming to efficiently perform their major functions of immunomodulation, phagocytosis, and antigen presentation (35, 37). Tissue resident macrophages are long-lived cells and thus rely on fatty acid oxidation and oxidative phosphorylation for both redox balance and prolonged survival (38–40). Conversely, proinflammatory macrophages upregulate glycolysis and exhibit two breaks in the TCA cycle at succinate dehydrogenase (SDH) and isocitrate dehydrogenase (IDH), leading to the accumulation of citrate and succinate (41–43). These metabolites increase flux through the pentose phosphate pathway and upregulate fatty acid synthesis to promote the synthesis of ROS and intermediates for proinflammatory cytokine production and cell proliferation (39, 44–46).

During the resolution of inflammation, macrophages accumulate glutamine from the extracellular environment or through the engulfment of apoptotic cells and break it down into the TCA cycle intermediate α-ketoglutarate (47, 48). The resulting upregulation of oxidative phosphorylation facilitates enhanced efferocytosis and production of the anti-inflammatory and pro-resolving cytokine, IL-10 (47, 49). Enhanced IL-10 signaling potentiates oxidative phosphorylation and inhibits glucose uptake, further suppressing glycolysis (50, 51). It should be noted that there is some controversy regarding the requirement of fatty acid oxidation for anti-inflammatory activation (52–58). Nonetheless, the clear reliance of pro-inflammatory macrophages on aerobic glycolysis and that of anti-inflammatory and tissue-resident cells on oxidative phosphorylation suggests that targeting metabolic reprogramming may be an efficacious strategy to modulate macrophage activity.

Recent studies suggest that crosstalk between macrophages and other immune cells such as mast cells may affect their metabolic fates and enhance functional outcomes during immune activity. Holter et al. demonstrated that IgE-activated mast cells can drive macrophage polarization, phagocytosis, and antibacterial activity by influencing metabolic and epigenetic processes in macrophages (59). Macrophages exposed to supernatants from IgE-activated mast cells exhibited increased basal oxygen consumption rates (representative of mitochondrial respiration), ATP production, maximal respiration, and spare respiratory capacity compared to cells exposed to supernatants from non-sensitized mast cells. This suggests that during inflammatory states such as allergic responses, crosstalk between immune cells may have the potential to regulate metabolic plasticity and function.

### 2.3 Myeloid-derived suppressor cells

Myeloid-derived suppressor cells (MDSCs) comprise pathologically activated neutrophils and monocytes that exhibit potent immunosuppressive activity (60). In cancer, MDSCs adapt to the anoxic tumor microenvironment by upregulating mechanistic target of rapamycin (mTOR) and HIF-1α signaling, as well as expression of lipid uptake scavenger receptors (e.g., CD36 and macrophage scavenger receptor 1 [MSR1]) and key fatty acid oxidation enzymes (e.g., CPT1A, acyl-CoA dehydrogenase medium chain [ACADM], hydroxyacyl-CoA dehydrogenase trifunctional multienzyme complex subunit alpha [HADHA], and peroxisome proliferator-activated receptor coactivator 1 beta [PGC-1β]) (61–64). This leads to a simultaneous increase in glucose uptake and glycolysis, as well as lipid uptake and oxidative phosphorylation that have both been shown to enhance the immunosuppressive activity of these cells (65–67).

### 2.4 Dendritic cells

Dendritic cells are professional antigen-presenting cells that link the innate to the adaptive immune system. They detect and proteolytically degrade pathogen-derived components, which are in turn loaded onto MHC class I or class II molecules to activate cytotoxic CD8+ T cells or helper CD4+ T cells, respectively (68). Dendritic cells encompass diverse subpopulations, including conventional cDC1s that are efficient at cross-presenting antigens to CD8+ T cells and cDC2s that specialize in CD4+ T cell activation (69). Plasmacytoid dendritic cells (pDCs) are critical in viral defenses by producing Type-I interferon, whereas monocyte-derived, inflammatory DCs (infDCs) regulate inflammation and infection (70). The differentiation and activation of dendritic cells are regulated by intracellular metabolism (71). At baseline, cDC1s are more heavily reliant on oxidative phosphorylation compared to cDC2s or pDCs, likely due to their larger mitochondrial mass and membrane potential (72–74). Treatment with LPS and poly(I:C) was shown to induce PI3K/AKT, nitric oxide (NO), and BNIP33 signaling that resulted in a rapid increase in glucose uptake and glycolytic flux, as well as a decrease in oxidative phosphorylation in cDC1s, cDC2s, pDCs, and infDCs (75–81). The requirement for this metabolic reprogramming in dendritic cell activity is evidenced by studies showing that inhibition of glycolysis with 2-deoxyglucose (2-DG) or knockdown of glycolytic inducers [e.g., HIF-1α and alpha enolase (ENO1)], impaired dendritic cell maturation, and subsequent T cell activation (78, 80, 82, 83). Similarly, in a model of allergic asthma, intranasal treatment with D-2-hydroxyglutarate suppressed allergic sensitization mediated by dendritic cells and promoted the activity of follicular regulatory T cells (84).

## 3 Steroid hormone regulation of inflammation

### 3.1 Glucocorticoids

The production of endogenous glucocorticoids is rapidly induced in response to inflammation and other stressors, but the secretion patterns are also affected by circadian and ultradian rhythms (85). Glucocorticoid receptors are expressed in essentially all immune cells in mice and humans where they exhibit time-, concentration-, cell type-, and in some cases, species-specific effects (86). Glucocorticoids bind to cytosolic glucocorticoid receptors and regulate gene expression by interacting with glucocorticoid response elements (GREs) in gene promoters. In addition, they induce transcription through protein-protein interactions in the cytosol and by composite binding to other transcription factors at DNA sequences containing GREs and a response element of a distinct transcription factor (85, 87). Glucocorticoids may also signal through membrane-bound glucocorticoid receptors to mediate rapid signaling (88). High concentrations or chronic administration of glucocorticoids promote apoptosis of immune cells, including T cells, macrophages, dendritic cells, and eosinophils in mice and humans (18, 89), whereas lower concentrations and shorter exposure windows exhibit more nuanced effects on inflammation and immunity.

In murine macrophages, glucocorticoids generally suppress production of pro-inflammatory eicosanoids (e.g., prostaglandins and leukotrienes), and pro-inflammatory cytokines (e.g., IL-1β, IL-6, TNFα, and IL-12), and they inhibit leukocyte migration (85, 90–94). Interestingly, expression of pro-inflammatory cytokines (e.g., IL-6 and TNFα) was significantly reduced in male rat livers compared to females after LPS administration, a response that correlated with enhanced survival (95). Conversely, the estrogen receptor antagonist ICI 182,780 was found to inhibit dexamethasone-induced reductions in TNFα, IL-1β, and iNOS in the lungs of rats treated with carrageenan (96).

Glucocorticoids also promote the resolution of inflammation by inducing expression of scavenger receptors (e.g., CD153, CD206, and MERTK), anti-inflammatory cytokines (e.g., TGF-β and IL-10), and the receptor for the pro-resolving lipid mediator, lipoxin A4, in human and murine macrophages, all of which potentiate macrophage phagocytosis and efferocytosis (97–101). Dichotomous effects have been observed in human T cells in which glucocorticoids suppress pro-inflammatory responses of Th1 and Th17 cells but promote the function of anti-inflammatory and immunosuppressive Th2 and Treg cells (85, 89, 102). Likewise, the expression of pro-inflammatory cytokines (e.g., IL-12 and TNFα) is reduced while that of anti-inflammatory cytokines (e.g., IL-10) is increased in dendritic cells treated with glucocorticoids (103). These effects on pro- and anti-inflammatory signaling and immune responses have resulted in their widespread use in asthma therapy and are associated with distinct changes in intracellular metabolism and metabolic reprogramming as described below (104, 105).

### 3.2 Androgens

Androgens are commonly known as male sex hormones but are produced by both sexes (106). The main androgen, testosterone, along with other androgens such as androstenedione, are produced by testicular Leydig cells, ovarian theca cells, and adrenal zona reticularis cells (107). Testosterone is locally converted into the more potent dihydrotestosterone (DHT) by 5α-reductase (108). Androgens signal by binding to the androgen receptor (AR), which interacts with androgen response elements (AREs) and recruits co-activators and co-repressors to regulate transcription of AR target genes. While no membrane-bound AR has been reported, androgens may mediate rapid, non-genomic effects by interacting with kinases such as SRC and PI3K in the cytoplasm (109). Androgen receptors are expressed by murine and human macrophages, monocytes, neutrophils, T cells, and B cells, in which they exhibit predominantly anti-inflammatory and immunosuppressive effects (17).

In human monocyte-derived macrophages, murine peritoneal macrophages, and human and murine macrophage cell lines (e.g., RAW 264.7 and J774A.1), testosterone dose-dependently inhibited LPS-induced production of nitric oxide (NO) and TNFα, as well as expression of inducible nitric oxide synthase (iNOS/*Nos2*) and TLR4 (110–115). Likewise, testosterone treatment increased production of anti-inflammatory IL-10 after LPS stimulation in a human macrophage cell line (111). Similar findings have been reported in T cells where androgens decreased murine and human T cell numbers and dampened the production of type I (T1) and type II (T2) inflammatory mediators (e.g., IFNγ, IL-4, IL-5) while enhancing production of immunosuppressive IL-10 (17, 116–121).

### 3.3 Progestogens

The main natural progestogen, progesterone (P4), is synthesized from cholesterol by the cholesterol side-chain cleavage enzyme and 3β-hydroxysteroid dehydrogenase (122). Progesterone signals by binding to progesterone receptors, which have two isoforms (A and B) that exhibit tissue-specific expression and functions (123). They exert agonistic and antagonistic effects on other steroid hormone pathways by interacting with other receptors, including estrogen receptors, and androgen receptor, glucocorticoid receptor, and mineralocorticoid receptor (123–126). Progesterone receptors are expressed by murine and human T cells, murine dendritic cells, human natural killer cells, and murine and human macrophages, where they generally suppress pro-inflammatory and enhance immunosuppressive activity (123, 127, 128).

For example, progesterone inhibited NF-κB signaling and reduced NO and IL-12 production in LPS-stimulated murine bone marrow-derived and alveolar macrophages, respectively (129–132). In this regard, the synthetic progestin, medroxyprogesterone acetate, triggered the differentiation of the human monocyte cell line, THP-1, into a more anti-inflammatory phenotype (e.g., upregulated CD163 and IL-10 expression) (133). In human, murine, and bovine CD4+ T cells, progesterone promotes Th2 and Treg expansion and activation (increased IL-4 and IL-10) and inhibits Th17 differentiation (reduced IL-2 and IL-12) (127, 134–138). Likewise, progesterone inhibited LPS and TLR-driven responses in human dendritic cells (e.g., reduced IL-1β, IL-6, and IFN-β); these immunosuppressive effects are important in ensuring successful pregnancies by preventing fetal rejection (128, 139–142).

### 3.4 Estrogens

Endogenous estrogens are steroid hormones derived from cholesterol (e.g., estrone [E1], 17β-estradiol [E2], estriol, estetrol [E4], and 27-hydroxycholesterol), which exhibit tissue- and life stage-specific production (143, 144). The different estrogens exhibit distinct pharmacology, with E2 exhibiting the greatest affinity for the estrogen receptors (ERs) (143, 144). Estrogens signal through myriad pathways, including genomic mechanisms mediated by two nuclear receptors (ERα and ERβ) that recruit various co-regulatory proteins and induce transcription at estrogen response elements (EREs), in addition to other promoters by tethering to transcription factors (e.g., AP-1 and NF-κB). Estrogens also signal through membrane-bound receptors (e.g., GPER1 and palmitoylated-ERα variants) to initiate non-genomic signaling, and these pathways often intersect and cross-regulate each other (145–148). ERα is the most widely expressed receptor isoform in immune cells, with positive expression in human and murine natural killer cells, T cells, neutrophils, eosinophils, dendritic cells, monocytes, and macrophages (113), while ERβ expression is less widespread and heavily debated (113, 149). GPER1 has been detected in human neutrophils and eosinophils, and palmitoylated ERα variants in natural killer cells, monocytes, and macrophages (113).

The effects of estrogens on immunity have been thoroughly reviewed by multiple groups who report cell- and disease-specific effects (19, 113). In acute inflammation models, E2 appears to enhance TLR-mediated pro-inflammatory responses in human and murine macrophages while also promoting T2 and anti-inflammatory activity in allergic asthma and cutaneous wound healing (110, 115, 150–157). The positive regulation of pro-inflammatory macrophage activity appears to be dependent on activation of STAT1, whereas the increased anti-inflammatory activation is driven by ERα-mediated phosphorylation and activation of STAT3 and interference with p65 subunit binding to the NF-κB complex (158–161). Estrogens modulate T cell activity throughout their entire life cycle, and similar discordant results have been reported in diverse T cell subpopulations. For example, E2 increased human and murine Th1 differentiation by inducing T-bet and enhancing IFNγ production while also promoting IL-4 production and favoring Treg expansion (162–169). These disparate results are likely due to the differences in the cellular microenvironment, cell type-specific utilization of coregulatory proteins, and concentration-dependent effects of estrogens (170–172). Several studies also suggest that estrogen may modulate human and murine mast cell behavior and activity during allergic diseases and other conditions such as endometriosis (173–175).

## 4 Immunometabolism as the link between steroid hormones and inflammation and immunity

### 4.1 Glucocorticoids

As discussed, glucocorticoid receptor signaling promotes anti-inflammatory and immunosuppressive activities of innate immune cells, including macrophages and myeloid-derived suppressor cells. Emerging evidence indicates that this is driven by metabolic changes. In a zebrafish model of hair cell ablation injury, activation of glucocorticoid signaling initiated a linear sequence of IL-10, polyamine, and IL-4 signaling, culminating in an increase in the expression of genes involved in oxidative phosphorylation facilitating wound repair (176). Other studies have shown that cortisol increased the expression of carnitine palmitoyltransferase II (CPT2) and glutaminase (GLS) in human monocyte-derived macrophages; this upregulated fatty acid oxidation and glutaminolysis, respectively, resulting in TCA cycle anaplerosis (177). In this regard, dexamethasone increased the expression of carnitine palmitoyltransferase I (CPT1) in human myeloid-derived suppressor cells, leading to an increase in fatty acid oxidation, mitochondrial respiration, and enhanced immunosuppressive activity (178). Collectively, these studies indicate that glucocorticoid receptor signaling promotes immunosuppression and the resolution of inflammation by enhancing oxidative phosphorylation in innate immune cells.

In addition, glucocorticoids have been found to indirectly increase mitochondrial respiration by inhibiting glycolytic activity. For example, treatment of macrophages (e.g., murine bone marrow-derived macrophages [BMDMs] and human THP-1 cells) and myeloid-derived suppressor cells with dexamethasone after exposure to E. coli, LPS, and ovalbumin (OVA) inhibited glycolysis and led to a compensatory increase in glutamine metabolism, anaplerosis, TCA cycle flux, and a restoration of basal and maximal oxidative phosphorylation (179–181). These studies highlighted reduced expression and inhibition of HIF-1α activity and subsequent reductions in expression of key glycolytic enzymes (e.g., Glut1, PDK, pyruvate kinase M2 [PKM2], lactate dehydrogenase a [LDH], and hexokinases) as major drivers of this metabolic plasticity (179, 180, 182). Of note, the shift toward oxidative phosphorylation was associated with reduced expression of T1 and T2 cytokines (e.g., IL-4, -5, -13, -1β), iNOS, and ROS production in these cells (179–181, 183). Thus, in addition to direct effects on oxidative phosphorylation, GR signaling downregulates HIF-1α, leading to a suppression of glycolysis and impaired pro-inflammatory activation; this results in a compensatory shift toward oxidative phosphorylation, enhancing anti-inflammatory and immunosuppressive activity.

The well-established immunosuppressive effects of glucocorticoids on T cells appear to also be mediated, in part, by changes in intracellular metabolism. Dexamethasone inhibited mRNA expression of glycolytic proteins [e.g., *Glut1*, *Glut3*, *Ldha*) in mouse and human T cells and leukemic cells (184, 185)]. This was associated with reduced pro-inflammatory gene expression (e.g., *Il2, Ifng*) (185). In Tregs, dexamethasone enhanced immunosuppressive activity by reducing glycolysis and increasing oxidative phosphorylation through miR-342-dependent inhibition of rapamycin-insensitive companion of mTOR (RICTOR) (186). It should be noted that the immunosuppressive effects of glucocorticoids are not always desirable. For example, the glucocorticoid, prednisolone, impaired the differentiation and effector function of memory CD8+ T cells by reducing glycolysis; this resulted in impairments in memory formation, recall antigen responses, and anti-tumor activity (187).

Conversely, glucocorticoids have also been shown to suppress oxidative phosphorylation; however, this appears to be a dose-dependent effect. For example, high-dose dexamethasone reduced mRNA and protein expression of mitochondrially encoded cytochrome c oxidase subunit I (MT-CO1) in murine macrophages and PKM2 expression in human chronic lymphocytic leukemia cells (188, 189). This was associated with reduced mitochondrial respiratory chain complex IV activity and loss of mitochondrial membrane potential, respectively, and increased apoptosis (188, 189). Thus, selectively regulating pro- or anti-inflammatory activity of immune cells may require targeting distinct metabolic pathways by careful titration of glucocorticoids.

### 4.2 Androgens

Although androgens do not appear to directly modulate glycolysis, they have been shown to regulate the expression of genes involved in oxidative phosphorylation. An analysis of previously published single-cell RNA-sequencing data collected from the prostates of castrated male mice identified reduced expression of complex (C) I, CIV, and CV subunits of the mitochondrial oxidative phosphorylation system in macrophage and T cell clusters. Of note, this response was reversed after administration of exogenous testosterone (190). In murine myeloid-derived suppressor cells, androgen receptor up-regulated mitochondrial pyruvate carrier 2 (MPC-2) expression to enhance TCA cycle flux, mitochondrial respiration, and immunosuppressive activities (191). Inhibition of the androgen receptor with enzalutamide reduced oxidative phosphorylation, which in turn resulted in a compensatory increase in GLUT1 expression, glucose uptake, and glycolytic flux (191). The authors suggested this metabolic plasticity led to persistent immunosuppressive activity and limited the effectiveness of anti-androgen therapy in prostate cancer (191). In a mouse model of allergic asthma, androgen receptor signaling was found to restrict allergen-induced airway inflammation and airway hyperresponsiveness by reducing glutamine uptake and glutaminolysis in CD4+ T cells by reducing the expression of glutamine uptake transporters (e.g., *Slc1a5* and *Slc38a1*) (192). The reduction in glutamine shunted metabolism away from the TCA cycle and decreased glutathione levels; this resulted in increased ROS production, which prevented Th17 differentiation and effector function by promoting a regulatory phenotype (192). Thus, the effects of testosterone on bioenergetics and inflammatory activity appear to depend on the cell type.

### 4.3 Progestogens

In general, progesterone seems to enhance the metabolic activity and function of myeloid cells. Gonadectomy of female rats reduced the production of hydrogen peroxide and phagocytic capacity of both unstimulated macrophages and those stimulated by phorbol myristate acetate (PMA). Progesterone supplementation reversed these effects, and this was associated with an increase in glutaminase activity (193). In immature human dendritic cells, progesterone increased expression of mitochondrial complex II-V genes (succinate dehydrogenase complex iron sulfur subunit B, [SDHB], succinate dehydrogenase complex subunit C [SDHC], ubiquinol-cytochrome C reductase complex III subunit VII [UQCRQ], cytochrome c oxidase subunit II [COX II], and ATP synthase F1 subunit alpha [ATP5A]), and mitochondrial pyruvate carrier 1 (MPC1) (194). In addition, genes involved in fatty acid oxidation (e.g., hydroxyacyl-CoA dehydrogenase trifunctional multienzyme complex subunit beta [HADHB], CPT1, and CPT2), as well as genes involved in fatty acid synthesis (e.g., acetyl-CoA carboxylase alpha [ACACA] and fatty acid synthase [FASN]) were upregulated (194). Another study by the same group found that progesterone also increased mRNA expression of glycolytic enzymes [e.g., HK2, LDHA, and pyruvate dehydrogenase (PDH)] in immature dendritic cells (194). This was associated with enhancements in parameters of oxidative phosphorylation (e.g., basal and maximal oxidative phosphorylation and spare respiratory capacity), as well as glycolytic capacity, and increased cell-surface expression of immunosuppressive inhibitor receptor Ig-like transcript 4 (ILT4) (194). The authors concluded that the progesterone-mediated enhancement of metabolic activity in immature dendritic cells helped maintain immune tolerance in the decidual microenvironment to prevent fetal rejection and ensure reproductive success.

### 4.4 Estrogens

Estrogens appear to differentially regulate intracellular bioenergetics, inflammatory, and immune responses in a cell type- and microenvironment-dependent manner. Spare respiratory capacity, differentiation, and activation were reduced in murine Th17 cells lacking ERα (195). Similarly, treatment of murine RAW 264.7 cells with E2 blunted LPS/IFNγ-induced reductions in basal oxidative phosphorylation, and this was associated with reduced TNF-α and increased IL-10 secretion compared to cells treated with LPS/IFNγ; however, no response was observed in murine BMDMs (196). It appears that the effects on mitochondrial function and inflammatory activity were mediated by E2-dependent increases in Sirt3 expression and mitochondrial protein acetylation (196). A key role for ERα was established in one study showing that murine BMDMs deficient in ERα exhibited reduced fatty acid oxidation compared to wild types (155). In contrast, other studies have found that E2 inhibited receptor activator of nuclear factor-κB ligand (RANKL)-induced upregulation of oxidative phosphorylation (197–199). The authors of the latter studies concluded that the reduced mitochondrial respiration led to enhanced apoptosis and postulated that this immunosuppressive effect of E2 explained why post-menopausal women exhibit increased osteoclast activity and bone resorption (197–199). Interestingly, these seemingly dichotomous effects of E2 on intracellular bioenergetics could be a result of differential receptor activation, as E2 was found to inhibit F0F1-ATP synthase activity via non-genomic mechanisms in a human osteoclastic cell line (FLG 29.1 cells) (200). In this regard, E2 rapidly increased lactate production in human MCF-7 breast cancer cells in a PI3K/AKT-dependent mechanism that was speculated to be initiated by a membrane-bound pool of ERα (201).

In murine alveolar macrophages, genetic deletion of ERα resulted in increased glycolytic capacity and glycolytic reserve with no effect on mitochondrial oxidative phosphorylation; these data suggest that ERα functions to suppress glycolysis (202). It appears this may be a result of the alveolar microenvironment, which is characterized by low glucose levels. One study found that in high glucose conditions, E2 dose-dependently increased lactate and glucose-6-phosphate (G6P) production in human MCF-7 breast cancer cells, whereas in low glucose conditions, E2 dose-dependently reduced lactate production and enhanced TCA cycle flux (201). These results indicate that the local fuel supply can dictate the effect of E2 on metabolic plasticity and may explain the observations of increased glycolysis in ERα-deficient murine alveolar macrophages. While estrogen also regulates mitochondrial DNA transcription, mitochondrial morphology, and mitochondrial fission and fusion in breast cancer cells (reviewed in 203), these mechanisms are understudied in the context of immune cells.

Estrogenic endocrine-disrupting chemicals also influence macrophage activity by modulating intracellular bioenergetics. Exposure of murine J774A.1 cells to the weakly estrogenic plasticizer, bisphenol S (BPS), increased levels of glycolytic metabolites (e.g., lactate, pyruvate, and glucose-6-phosphate) and increased mRNA and protein expression of glycolytic enzymes (e.g., PKM2, LDHA, hexokinase 1 [HK1], and HK2). The BPS-induced enhancements in glycolytic activity were associated with increased expression of pro-inflammatory (e.g., TNF-α, IL-1β, and IL-6) and reduced expression of anti-inflammatory (e.g., TGFβ and IL-10) cytokines and growth factors (204). Another study found that the analogous plasticizer, bisphenol F (BPF), dose-dependently increased expression of glycolytic enzymes (e.g., HIF-1α, GLUT1, PKM2), lactate production, and glucose consumption, and increased the secretion of pro-inflammatory cytokines (e.g., TNF-α, IL-1β, and IL-6) in murine RAW 264.7 cells; this was confirmed to be through an ERα and PI3K/AKT-dependent mechanism (205). These studies highlight environmental estrogens as immunomodulatory agents and demonstrate the critical role of metabolic plasticity in pro- and anti-inflammatory macrophage activation.

## 5 Discussion

**Figure 1:**
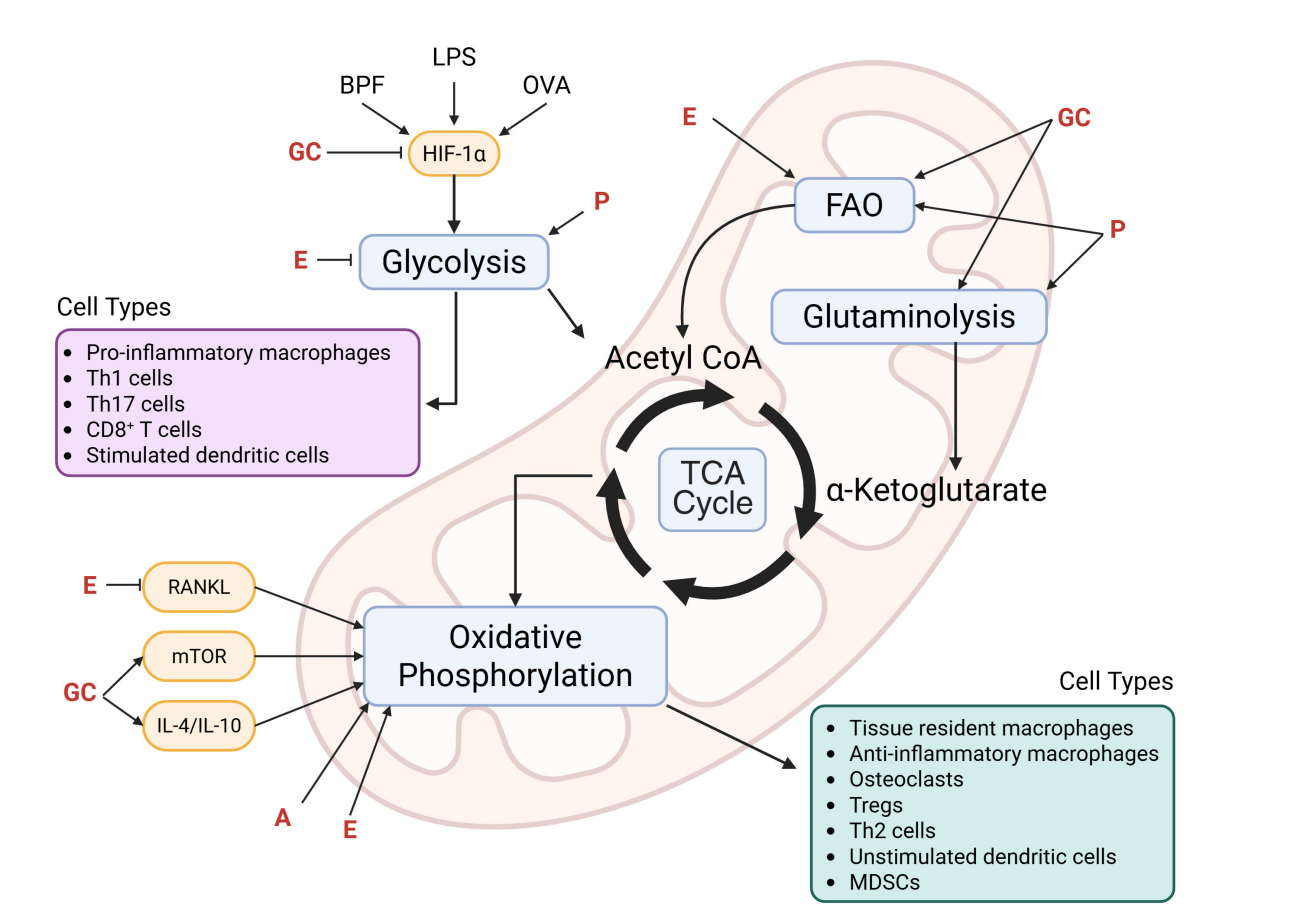
Immunometabolic targets of steroid hormones. Activated cells (e.g., pro-inflammatory macrophages, Th1, Th17, CD8^+^ T cells, and stimulated dendritic cells) preferentially rely on glycolysis and exhibit dysregulation of the TCA cycle. This rapidly generates glycolytic intermediates that can be used for cell proliferation and production of ROS and inflammatory mediators. On the other hand, immunomodulatory cells (e.g., anti-inflammatory macrophages, Tregs, Th2 cells, and myeloid-derived suppressor cells [MDSCs]), tissue resident cells (e.g., macrophages and osteoclasts), and unstimulated dendritic cells, are long-lived and rely on the more efficient oxidative phosphorylation pathway to satisfy their energy requirements. Key mediators (e.g., hypoxia-inducible factor 1-alpha [HIF-1α], receptor activator of nuclear factor-κB ligand [RANKL], mechanistic target of rapamycin [mTOR], interleukin [IL]-4, and IL-10) that are known to be activated by inflammatory stimuli (e.g., bisphenol-F [BPF], lipopolysaccharide [LPS], and ovalbumin [OVA]) and/or modulated by steroid hormones are depicted in yellow, and the immunometabolic pathways they target are shown in blue. Of note, some steroid hormones exhibit dichotomous regulation driven by cell type-, microenvironment-, and concentration-dependent mechanisms as described in the review. GC, glucocorticoids; E, estrogens; A, androgens; P, progestogens; FAO, fatty acid oxidation. Created in BioRender. Smith, C. (2025) https://BioRender.com/su4w0te.

Immune cells undergo metabolic reprogramming to meet the energetic and biosynthetic demands of their activation, differentiation, and effector functions. This reprogramming involves dynamic shifts between glycolysis, the TCA cycle, and oxidative phosphorylation and is influenced by both intrinsic signaling pathways and extrinsic factors such as steroid hormones. Steroid hormones—including glucocorticoids, androgens, progestogens, and estrogens—regulate immune responses by modulating intracellular metabolism in rodents and humans, although species-specific effects have been reported. The effects of steroid hormones can also vary depending on the microenvironment and disease-state. Nonetheless, these findings underscore the intricate and bidirectional relationship between metabolism and immune regulation and highlight steroid hormones as key modulators of immunometabolic activity across multiple immune cell types (**Figure 1**).

While not the focus of this review, it should be noted that the adrenal-derived steroid hormone and androgen and estrogen precursor, dehydroepiandrosterone (DHEA), regulates intracellular metabolism and inflammatory activity. It has been found to inhibit glucose-6-phosphate dehydrogenase and the pentose phosphate pathway in human and mouse endometrial stromal cells and human breast cancer cells (206, 207), and to increase oxidative phosphorylation in cumulus cells of aged infertile patients and in liver mitochondria of developing rats (208, 209). Although it is not clear if DHEA modulates bioenergetic pathways in immune cells, it has been shown to attenuate LPS-induced production of pro-inflammatory mediators in murine RAW 264.7 macrophages (210); thus, future investigators into a role for this hormone in regulating immunometabolism are warranted. In conclusion, defining the cell type-, microenvironment-, disease-state, and concentration-dependent effects of steroid hormones on metabolic reprogramming in immune cells will facilitate more precise therapeutic targeting of immune cell activity to mitigate inflammatory- and immune-mediated diseases.
